# The Effect of Different Foundation Materials on the Color of Monolithic Zirconia at Different Thicknesses

**DOI:** 10.30476/DENTJODS.2021.85516.1131

**Published:** 2021-12

**Authors:** Elham Ansarifard, Mina Mohaghegh, Maryam Pakniyat Jahromi, Rashin Giti

**Affiliations:** 1 Dept. of Prosthodontics, School of Dentistry, Shiraz University of Medical Sciences, Shiraz, Iran; 2 Student, School of Dentistry, Shiraz University of Medical Sciences, Shiraz, Iran

**Keywords:** Color, Thickness, Foundation, Ceramics, Zirconium, Gold Alloys

## Abstract

**Statement of the Problem:**

Monolithic zirconia restoration has been introduced to overcome the porcelain chipping. Different factors can affect the color of monolithic zirconia, so achieving the desired color in the restorations is considered as a challenge.

**Purpose:**

The purpose of this *in vitro* study was to determine the effect of different foundation materials on the color of monolithic zirconia at different thicknesses.

**Materials and Method:**

In this experimental study, thirty ceramic disks in three thicknesses (i.e. 0.6mm, 1.1mm and 1.5mm) were fabricated from high translucency shade A_2_ monolithic zirconia block. Disk shaped foundation materials were fabricated from nickel chromium alloy (Ni-Cr), non-precious gold alloy (NPG), zirconia, and shade A_2_ composite resin. The color was measured by a spectrophotometer. The color differences (∆E) in the control and the test groups were calculated. The data were analyzed using two way ANOVA and compared with the posthoc Tukey test (a=0.05).

**Results:**

Ceramic thickness and foundation materials had a significant effect on the mean values of ∆E of monolithic zirconia ceramics (*p*= 0.001). The highest amount of ∆E value was observed in NPG, while Ni-Cr resulted in the lowest ∆E. Unacceptable results (∆E>2.25) were observed for monolithic zirconia ceramics on NPG foundation material with a thicknesses of 0.6 and 1.1mm. The mean L^*^ values of all foundation materials were higher than those of the control group except for Ni-Cr. The highest a^*^ was seen in NPG and the mean b^*^ values of all tested foundation materials were higher than those of the control group except for Ni-Cr.

**Conclusion:**

Increasing the thickness of monolithic zirconia decreased the color mismatch. High translucent monolithic zirconia could mask the color of Ni-Cr and zirconia in all three thicknesses (∆E<2.25), while it could not mask the color of NPG under thickness of 1.5mm.

## Introduction

The main challenge in esthetic dentistry is to optimally match the optical properties of restorative materials with the natural teeth [ 1 - 10 ]. Different ceramic systems are commercially available now [ 2 , 11 ]. Among different types of ceramics, the use of zirconia restorations is considerably increasing. Improved physical, mechanical and chemical properties, high fracture resistance and flexural strength, and excellent biocompatibility are some advantages of this ceramic [ 4 , 12 - 15 ]. Although fracture resistance of these prostheses is high, chipping of porcelain, veneer is a major complication. One solution to overcome this problem, was introduction of translucent full anatomical monolithic zirconia restorations [ 12 - 13 , 16 ].

Different factors can influence the final color of a ceramic restoration; thus, achieving the desired final color in these restorations is considered as a challenge. These factors include the degree of opalescence [ 17 - 19 ], translucency [ 17 - 20 ], fluorescence [ 17 - 19 ], condensation technique, shape properties, surface texture [ 18 - 19 ], chemical nature of the ceramic [ 17 ], ceramic brand [ 18 , 21 ], batches [ 18 , 22 ], underlying tooth structure color [ 2 - 3 , 18 , 20 , 23 ], ceramic firing temperature [ 20 , 22 ], number of ceramic firing cycles [ 18 , 22 ], surface glaze [ 20 , 22 ], ceramic thickness [ 2 , 18 , 22 ], color of the cement [ 2 , 3 , 17 - 18 , 20 , 23 ], manufacturer, and the type of the substructure [ 3 , 17 , 21 - 24 ].

One of the most important features for ceramic selection is the translucency of the material. Unlike high-strength core ceramics, high translucent ceramic systems show better esthetic results because they permit more light to transmit and scatter. Light transmission is not always an advantage; this means that with increasing translucency, their masking ability reduces, so they are more prone to showing their underlying structures like discolored tooth, foundation materials or luting agents [ 2 - 3 , 25 - 26 ]. Semi-translucent zirconia structure permits a little light to enter and scatter, so it might be concluded that the underlying tooth or substructure has an influence over the resulting color [ 27 ]. 

To fabricate the ceramic restorations that are more similar to natural dentition in terms of optical properties, the capability of the restoration to mask color variations present in the underlying substructure should be recognized [ 18 ]. 

Several studies have evaluated the effect of ceramic thickness on masking the color of different foundation materials [ 2 , 4 - 5 , 11 , 18 , 28 - 29 ]. As shown in a study, lithium disilicate glass-ceramic and leucite-based glass-ceramic with a thickness of 2.5 mm could mask the color of yellow zirconia. Zirconia-reinforced lithium silicate glass ceramic with a thickness of 2.5mm could cover the color of yellow zirconia and titanium [ 4 ]. Another study indicated that a 1 mm lithium disilicate ceramic had the ability to mask the gold foundation [ 5 ], while another study showed that a 1.6 mm leucite-based heat-pressed ceramic could cover the color of gold [ 11 ]. The results of one research demonstrated that lithium disilicate with a thickness of 1.5mm could cover the color of silver-palladium and could mask the color of composite resin with a thickness of 2mm [ 5 ].

In general, these studies suggested that by increasing the ceramic thickness, shade matching is improved [ 4 - 5 , 11 ]. Although many studies reported the masking ability of different types of ceramic systems, limited information is on hand regarding the masking ability of monolithic zirconia [ 2 , 4 - 5 , 11 , 18 , 28 - 29 ]. The purpose of this study was to find out the influence of different foundation materials on optical properties of monolithic zirconia at variable thicknesses. The null hypothesis was that the foundation materials and ceramic thickness would not affect the final color of monolithic zirconia restoration.

## Materials and Method

**Figure 1:**
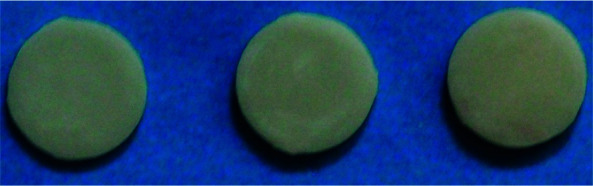
Monolithic zirconia disk specimens (from left to right: ceramic thicknesses of 0.6, 1.1, and 1.5 mm)

Thirty ceramic disks with shade A_2_ were cut from high translucent monolithic zirconia block (Kerox dental zirconia). The specimens had thicknesses of 0.6mm, 1.1mm and 1.5mm (n=10) and diameter of 10mm )Table 1). A CAD/CAM system (IMES-ICORE CORITEC 340 i) milled the monolithic zirconia blanks to fabricate the specimens (Fabricating the specimens was done by dry-milling with four axes). Crystallization of the ceramics was done according to the manufacturer's recommendation in a furnace (MIHM-VOGT; Dental- Gerätebau, HT speed) in the temperature of 1450^°C^ for 8 hours. The specimens were then polished with 220, 400, 600, 800, 1000 and 1200-grit abrasive silicon carbide paper on a grinder-polisher machine (Phoenix Beta; Buehler) at100 rpm for 15 seconds under cooling water until the desired thickness (0.6, 1.1 and 1.5mm) had been achieved with a tolerance of ±0.02mm. The thickness of each specimen was controlled by a digital caliper (Mitutoyo Corp. CD-8" CSX; 500-197-20). The ceramics were cleaned in an ultrasonic bath (Elmasonic S-30; Dentec, North Shore) containing 98% ethanol for 15 minutes and finally dried with oil-free compressed air (Figure 1).

**Table 1:** The materials used

Material	Manufacturer	Type
Monolithic zirconia	Kerox dental zirconia	High translucent Shade H_2_
Composite resin	Nexco, Ivoclar Vivadent	Dentin Shade A_2_
Non- precious gold alloy	AalbaDent	Type 2; Cu 80.7% Al 7.8% Ni 4.3% Fe, Zn, Mn
Nickel- chromium alloy	4 all, Ivoclar vivadent	Ni 61.4% Cr 25.7% Mo 11% Si 1.5% Mn< 1% Al< 1% C< 1%
Zirconia	Dental Direkt GmbH	DD Bio ZW iso White

**Figure 2:**
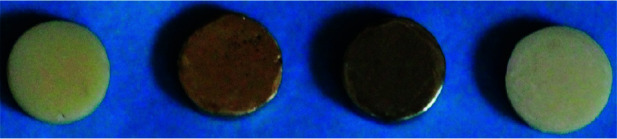
Foundation material specimens (from left to right: composite resin, non- precious gold alloy, nickel chromium alloy, and zirconia)

Four disk shaped foundation specimens were fabricated of nickel-chromium alloy (Ni-Cr) (4 all; Ivoclar vivadent), non-precious gold alloy (NPG) (AalbaDent), zirconia (DD Bio ZW iso; White, Dental Direkt GmbH), and shade A_2_ build up resin composite (dentin; Nexco, Ivoclar vivadent) (Table 1). The zirconia and wax patterns of Ni-Cr and NPG specimens were milled (12×3mm) by the same CAD/CAM system described earlier; then, casting of metallic specimens was done. Build up composite resin specimen (12×5 mm) was prepared in a mold pattern made from mixed polyvinyl siloxane impression material (Extrude medium body; Kerr). Composite resin was applied into the mold and a microscope slide was placed on the top of the mold. Then it was cured by a light polymerizing device (Lummat 100; Ivoclar vivadent) for 90 seconds. Composite resin specimen was considered as the control group and comparison of color differences was made with this group (Figure 2).

Monolithic zirconia disks were placed over each foundation material with a water drop between them to prevent the light refraction. Ceramic-foundation material assemblies were placed on a white background and color measurements were done by a spectrophotometer (VITA Easyshade^®^ V) in a dark room. For each ceramic-foundation material combination, five shade measurements were made and the mean value for each combination was calculated. The CIE L^*^a^*^b^*^parameters of each combination were recorded. In addition, the color difference (∆E) values between each ceramic-substructure assembly and the control group were calculated, using the following formula [ 2 , 14 , 17 - 18 , 27 - 32 ]:

$\mathrm{∆E}={\left[{\left(L_{T}-L_{C}\right)}^{2}+{\left(a_{T}-a_{C}\right)}^{2}+{\left(b_{T}-b_{C}\right)}^{2}\right]}^{1/2}$

Where T represents the test groups (NPG, Ni-Cr and zirconia) and C represents the control group which was the composite resin. In this study, ∆E> 1.3 was set as clinically perceptible and ∆E< 2.25 was considered as clinically acceptable. The ability of monolithic zirconia specimens to mask the underlying structure was defined by the clinically acceptable threshold (∆E= 2.25); it means with color differences below 2.25, monolithic zirconia ceramic could mask its underlying material [ 3 ].

The normality assumption was assessed using Kolmogorov-Smirnov test. The data were statistically analyzed using two-way ANOVA with a= 0.05 as the level of significance and whenever a significant interaction was observed, the post hoc Tukey test was carried out. All the computational work was done using the statistical software (IBM SPSS Statistics v18.0; IBM Corp). 

## Results

**Figure 3:**
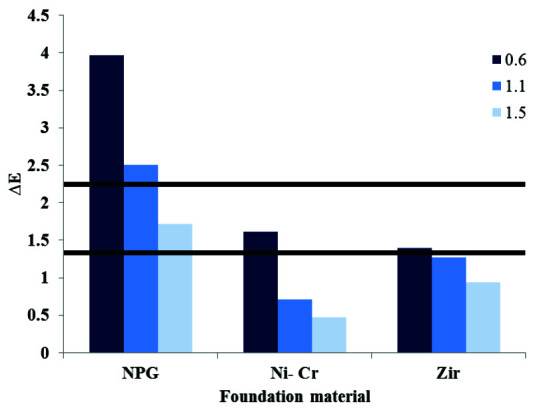
Mean ∆E values of the test groups at different thicknesses. Lines indicate perceptibility threshold (∆E= 1.3) and clinical acceptability threshold (∆E= 2.25)

Kolmogorov-Smirnov test revealed no violation from normal distribution in the groups. As shown in Table 2, ceramic thickness, foundation materials, and interaction of these two variables had a significant effect on the mean values of ∆E of monolithic zirconia ceramic assemblies (*p*= 0.001). The mean values of ∆E are shown in Table 3 and Figure 3.

**Table 2:** Results of a two-way ANOVA for ∆E value

Source	SS	df	MS	F	*p* value
Foundation	39.60	2	19.80	95.74	<0.001
Thickness	17.85	2	8.93	43.17	<0.001
Interaction	6.25	4	1.56	7.56	<0.001
Error	11.17	54	0.21	-	-
Total	241.68	63	-	-	-

SS: Type III sum of square, MS: Mean square, df: degrees of freedom

**Table 3:** Comparison of mean ∆E between different ceramic thickness and foundation materials

Foundation	Thickness (mm)			*p* Value*
	0.6	1.1	1.5	
NPG	3.97±0.82 ^A, a^	2.51±0.18 ^A, b^	1.72±0.21 ^A, c^	<0.001
Ni Cr	1.61±0.68 ^B, a^	0.71±0.23 ^B, b^	0.47±0.28 ^B, b^	<0.001
Zirconia	1.40±0.68 ^B, a^	1.27±0.10 ^C, a^	0.94±0.12 ^C, a^	0.113
*p* value*	<0.001	<0.001	<0.001	<0.001

The values in the table are mean±SD.

* One-way ANOVA F test.

Within columns, the same upper letter indicates the insignificant differences between the mean values of foundation materials (Tukey HSD test).

Within rows, the same lower letter indicates the insignificant difference between the mean values of ceramic thicknesses (Tukey HSD test).

The highest amount of ∆E value was for NPG, followed by zirconia, while Ni-Cr resulted in the lowest ∆E (Table 3). Multiple comparisons showed that the ∆E values of NPG with the ceramic thicknesses of 0.6, 1.1 and 1.5 mm were significantly higher than those in the other test groups (*p*< 0.05) (Table 3). The ∆E values of Ni-Cr with the ceramic thicknesses of 1.1 and 1.5mm were significantly lower than those in the other test groups. At 0.6mm, there was no significant difference between mean ∆E of zirconia and Ni-Cr (*p*< 0.05) (Table 3). For NPG, significant differences were seen in the mean values of ∆E among the three ceramic thickness groups (Table 3). For Ni-Cr, significant differences existed in the mean values of ∆E between ceramic thicknesses of 0.6 and 1.1, 0.6 and 1.5 (Table 3). For zirconia, among all the three ceramic thicknesses there were no significant differences in the mean values of ∆E (Table 3).

For ceramic thickness of 0.6mm, there were significant differences in the mean values of ∆E between NPG and zirconia, NPG and Ni-Cr (Table 3). For ceramic thicknesses of 1.1mm and 1.5mm, significant differences were observed in the mean values of ∆E among all the three foundation materials (Table 3). 

The mean color differences of NPG with ceramic thicknesses of 0.6 and 1.1mm were above the clinically acceptable threshold (∆E> 2.25). For ceramic thickness of 1.5mm, the mean color difference of NPG was higher than the perceptible threshold, but it was clinically acceptable (1.3< ∆E< 2.25). The mean color differences of Ni-Cr and zirconia with a thickness of 0.6 mm were clinically perceptible (∆E> 1.3) and with ceramic thicknesses of 1.1 and 1.5mm they were not perceptible (∆E< 1.3) (Table 3 and Figure 3). The results of a Two-way ANOVA and the mean values of L^*^, a^*^ and b^*^ values are given in Table 4 and 5, respectively.

**Table 4:** Results of a two-way ANOVA for L^*^, a^*^, and b^*^ values

Variable	Source	SS	df	MS	F	*p* Value
	Foundation	22.38	3	7.46	3.25	0.027
	Thickness	32.01	2	16	6.98	0.002
L	Interaction	2.03	6	0.34	0.15	0.989
	Error	162.73	71	2.29	-	-
	Total	619862.55	83	-	-	-
	Foundation	30.01	3	10	458.28	< 0.001
	Thickness	3.69	2	1.84	84.46	< 0.001
A	Interaction	2.9	6	0. 48	22.13	< 0.001
	Error	1.57	72	0. 02	-	-
	Total	273.5	84	-	-	-
	Foundation	99.74	3	33.25	11.82	< 0.001
	Thickness	4.76	2	2.38	0.85	0.433
B	Interaction	19.72	6	3.29	1.17	0.333
	Error	202.49	72	2.38	-	-
	Total	61007.77	84	-	-	-

SS: Type III sum of square, MS: Mean square, df: degrees of freedom

**Table 5:** Mean (±SD) CIE Lab values of specimen assemblies with different ceramic thickness and foundation material

Thickness/ foundation material	L^*^	a^*^	b^*^
Composite			
0.6 mm	86.38± 2.49	1.31± 0.09	26.27± 2.53
1.1 mm	86.42± 0.50	1.22± 0.09	26.37± 0.71
1.5 mm	85.25± 0.38	1.18± 0.12	26.84± 1.05
Total	86.02± 1.51	1.24± 0.11	26.49± 1.57
NPG			
0.6 mm	87.31± 2.33	3.41± 0.33	29.47± 1.76
1.1 mm	87.08± 0.69	2.54± 0.07	28.38± 0.54
1.5 mm	85.77± 0.52	2.15± 0.12	28.12± 0.96
Total	86.70± 1.56	2.70± 0.57	28.66± 1.28
Ni-Cr			
0.6 mm	85.98± 2.91	1.57± 0.22	24.82± 3.09
1.1 mm	86.27± 0.68	1.35± 0.07	25.72± 0.72
1.5 mm	85.17± 0.45	1.22± 0.11	26.57± 1.27
Total	85.81± 1.72	1.38± 0.20	25.71± 2.01
Zirconia			
0.6 mm	87.68± 2.13	1.52± 0.07	26.04± 2.84
1.1 mm	87.64± 0.49	1.30± 0.08	26.60± 0.67
1.5 mm	85.94± 0.59	1.25± 0.11	27.28± 1.04
Total	87.09± 1.49	1.36± 0.14	26.64± 1.77

As shown in Table 5, For L^*^ and b^*^ values, there was no significant interaction effect between thickness and foundation. Mean L^*^ was significantly higher in zirconia compared to Ni-Cr (p= 0.038). However, no significant differences were found between other foundations. Moreover, mean L^*^ was significantly lower in thickness 1.5mm compared to 0.6 and 1.1mm. For b^*^ value, NPG had higher values compared to other foundations (*p*< 0.05). However, mean b^*^ was not significantly different between the three thicknesses (*p*= 0.433). For a^*^ value, interaction effect was significant (*p*< 0.001).

Subgroup analyses revealed that for all foundations, mean a^*^ was higher for thickness 0.6mm compared to the two other thicknesses. In all thickness values, NPG had the greatest mean a^*^ compared to other foundations. Moreover, in thickness 1.1, zirconia had higher mean a^*^ compared to composite. 

In order to assess the sufficiency of the sample size used in our experiments, power analysis was performed over the investigated data set. This analysis showed that the power values were >80% for all cases where the amount of effect size was at least moderate (ES ≤ 0.60). This result confirms that the amount of sample size has been sufficiently large.

## Discussion

The result of the present study showed that the foundation material, thickness, and interaction of these variables had a significant effect on the optical properties of monolithic zirconia (Table 2); therefore, the null hypothesis was rejected.

Previous studies evaluated the masking ability of various ceramic systems [ 3 - 5 , 11 , 17 , 24 , 29 - 31 ], but to the best of our knowledge, the masking ability of monolithic zirconia had not been reported before. 

The calculation of a color difference between two objects does not have a clinical meaning without determining the parameters that have some practical implications. Therefore, determining the significance of color changes by assessing the value that is visually detected (perceptibility threshold) and the value of color difference between the teeth and esthetic dental restorations that most individuals would consider unacceptable (acceptability threshold) is important [ 8 , 32 ]. Different studies considered different thresholds of ∆E for clinical acceptability and perceptibility [ 3 - 5 , 17 , 33 ]. Ghinea *et al*. [ 33 ] evaluated the color difference thresholds in dental ceramics. They showed that the mean 50:50% acceptability and perceptibility thresholds obtained with the best fit were ∆E00= 2.23 and ∆E00= 1.25, respectively. Dede *et al*. set ∆E= 1.3 as the perceptual threshold and set ∆E< 2.25 as clinically acceptable [ 3 ]. Jirajariyavej *et al*. [ 4 ] considered ∆E<3 as clinically acceptable. Pires *et al*. [ 15 ] and Niu *et al*. [ 17 ] considered values of ∆E<5.5 as clinically acceptable and ∆E>2.6 as clinically perceptible. In our study, ∆E=1.3 was set as the perceptual threshold and ∆E<2.25 was set as clinically acceptable.

According to the results of the present study, the masking ability of monolithic zirconia depends on the foundation material and ceramic thickness. Color difference caused by NPG was perceptible in all the three ceramic thicknesses (∆E> 1.3) and it was clinically unacceptable with thicknesses of 0.6 and 1.1 mm (∆E> 2.25) (Table 3 and Figure 3). As a result, in a clinical case of view in application of NPG as a foundation material and high translucent monolithic zirconia as a crown, tooth reduction should be at least more than 1.1 mm to mask the underlying NPG. It is also recommended to determine the shade of the foundation, using a stump shade guide and report the shade to the laboratory, taking characterization into consideration to counsel the adverse effect of foundation shade on the final esthetic outcome. The color differences caused by Ni-Cr and zirconia with a ceramic thickness of 0.6mm were above the perceptible threshold, but they were clinically acceptable. For thicknesses of 1.1 and 1.5mm, the color differences were not perceptible (Table 3 and Figure 3). In a clinical situation, if the clinician intends to mask the color of Ni-Cr or zirconia by high translucent monolithic zirconia, the tooth reduction of more than 0.6 mm could be recommended. According to the literature, masking the silver to gray hue is harder than the gold to yellow one by ceramic restorations [ 34 ]. However, this research indicated that the use of monolithic zirconia led to a different result in masking ability. Further studies are required to assess various kinds of monolithic zirconia with different translucencies, thicknesses, surface characterizations, shades, and other factors. Previous studies reported suitable ceramic thicknesses and foundation materials to be masked by different ceramic types [ 4 - 5 ]. Jirajariyavej *et al*. [ 4 ] concluded that the ceramic thickness of 2.5 mm and the use of yellow shaded zirconia abutment were suitable to be covered by high translucent glass ceramic (∆E< 3). Niu *et al*. [ 5 ] showed that lithium disilicate ceramic with a thickness of 1 and 1.5mm could not cover composite resin. It also could not mask silver- palladium (Ag-Pd) with thickness of 1mm and its result was clinically unacceptable (∆E> 5.5).They concluded that ∆E value of a ceramic thickness of 2 mm was clinically acceptable for covering composite resin.

Based on our results, the mean L^*^ values of all foundation materials were higher than those of the control group was, except for Ni-Cr (Table 5). This may be due to the grayish shade of Ni-Cr. NPG and zirconia increased the lightness of the restoration, while Ni-Cr decreased its lightness. Zirconia had the highest L^*^ value (Table 5). This may be related to the white color of zirconia used in this study, so it increased the whiteness of final restoration more than other materials. However, there was no significant difference in the L^*^ value between zirconia and the control group (Table 5). Similarly, Dede *et al*. [ 3 ] reported that among different foundation materials (Titanium, Gold-palladium, and zirconia), zirconia had the highest L^*^ value when different types of ceramics were used (a heat-pressed lithium disilicate ceramic with a core translucency of medium opacity and high translucency, a glass infiltrated magnesium aluminate, and a Y-TZP ceramic). In addition, the mean L^*^ value of zirconia was higher than that of the control group that was shade A_2_ composite resin. Moreover, Oh and Kim [ 29 ] found that in three types of zirconia systems (Lava, Cercon, and Zirkonzahn) with two ceramic thicknesses (1 and 1.5 mm), the mean L^*^ value of gold alloy was higher than Ni-Cr and the control group that was shade A_2_ composite resin. In contrast to the present study, Tabatabaian *et al*. [ 31 ] showed that for zirconia crown at a thickness of 0.5 mm, the mean L^*^ value of foundation materials NPG (78.98), zirconia (82.16) and Ni-Cr (78.24)) was lower than that of the control group (a white Teflon material (88.35)). This contradiction could be related to the differences in the internal structure, brand and thickness of different materials such as the ceramic and foundation materials used in these studies. However, these findings are also likely to be affected by the differences in the control groups.

The mean a^*^ value of all the materials were higher than the control group. The highest a^*^ value was observed in NPG (Table 5). As a result, NPG shifts the color of the final restoration to redness. Similarly, Oh and Kim [ 29 ] showed that among the foundation materials (Ni-Cr, gold alloy, and composite resin), gold had the highest a^*^ value in all the three ceramic systems and at all two ceramic thicknesses. Niu *et al*. [ 5 ] reported that in machinable lithium disilicate ceramics with three different thicknesses (1, 1.5 and 2mm) gold increased the mean a^*^ value more than silver-palladium and composite resin in all thicknesses. Tabatabaian *et al*. [ 31 ] showed that there was an increase in a^*^ value in all of the groups (NPG, zirconia and Ni- Cr). Moreover, they concluded that zirconia had the highest a^*^ value. These differences might be due to application of different brands of materials used in these two studies. Given the result of our research, it can be concluded that NPG shifts the color of ceramic restorations toward redness significantly. Therefore, for chroma adjustment, it could be recommended that complementary colors should be added and for hue adjustment, adding yellow color could decrease the red content of the yellow-red shade. The results of this study showed that the mean b^*^ values of all tested foundation materials were higher than those of the control group, except for Ni-Cr (Table 5). This might be the result of the yellow color tendency of NPG [ 31 ]. The highest mean b^*^ value was observed for NPG and its difference from the control group was significant (Table 5). Dede *et al*. [ 3 ] concluded that for zirconia as a ceramic, gold-palladium had the highest b^*^ value, but for lithium disilicate, zirconia had the highest b^*^ value. Tabatabaian *et al*. [ 31 ] showed that the mean b^*^ values of all tested foundation materials were higher than control group except for Ni-Cr, but the highest b^*^ value was recorded for zirconia.

The limitations of this in vitro study included the use of only one monolithic zirconia type in one shade and one translucency. Also, application of luting agent might affect the optical properties of monolithic zirconia ceramic which was not evaluated in this study. Considering the increase in the use of dental implants, it is worth determining the effect of other foundation materials including the current implant abutment materials on the masking ability of monolithic zirconia. Further studies are required to determine the effects of different shades, brands, translucency, and surface characterization of monolithic zirconia and luting agents on optical properties of monolithic zirconia.

## Conclusion

Based on the results of this in vitro study, the following conclusions were reached:

1. In fabricating monolithic zirconia crowns, it is essential to consider the ceramic thickness and type of foundation material simultaneously. By increasing the thickness of monolithic zirconia ceramic, the color difference decreased among all of the foundation materials.
2. NPG had the highest ∆E value with the ceramic thickness of 0.6 mm.
3. Regarding the semi-translucency characteristics of monolithic zirconia, unacceptable clinical outcomes results were observed for monolithic zirconia ceramic at thicknesses of 0.6 and 1.1 mm with NPG foundation material (∆E> 2.25).

## Acknowledgement

This research was supported by Shiraz University of Medical Sciences. We specially thank the statistics advisor of the research, Dr. Mehrdad Vossoughi who provided insight and expertise in statistical assignments and data analysis and greatly assisted the research and improved the manuscript. The study was supported in part by the vice-chancellery of Shiraz University of Medical Sciences, grant # 15422.

## Conflict of Interest

The authors declare that they have no conflict of interest.
